# Influence of *Lonicera japonica* and *Radix Puerariae* Crude Extracts on the Fecal Microbiome and Nutrient Apparent Digestibility of Finishing Pigs

**DOI:** 10.3390/ani12162109

**Published:** 2022-08-17

**Authors:** Zhonghao Liu, Ning Li, Zi Zheng, Chunhua Zhang, Zhengqun Liu, Chunling Song, Jun Yan, Shuqin Mu

**Affiliations:** 1 Institute of Animal Husbandry and Veterinary Medicine, Tianjin Academy of Agricultural Sciences, Tianjin 300381, China; 2 Beijing Tianfulai Biological Technology Co., Ltd., Beijing 102206, China

**Keywords:** finishing pigs, feces microbiota, *Lonicera japonica* crude extracts, *Radix Puerariae* crude extracts, nutrient apparent digestibility

## Abstract

**Simple Summary:**

This study evaluated the effects of *Lonicera japonica* and *Radix Puerariae* crude extracts as feed additives on finishing pigs. The results indicated diets supplemented with *L. japonica* and *Radix Puerariae* crude extracts improved growth performance, abundance of beneficial bacteria in feces, and digestibility of crude protein and total phosphorus in finishing pigs. These results suggest that *Lonicera japonica* and *Radix Puerariae* crude extracts could be a good feed additives for finishing pigs feeding.

**Abstract:**

This study aims to investigate the influence of adding *Lonicera japonica* (*L. japonica*) and *Radix Puerariae* crude extracts and their mixture to the diet of finishing pigs on their fecal microbes and nutrient apparent digestibility. A total of 72 healthy Duroc × Landrace × Yorkshire crossbred barrows without significant differences in body weight (93 ± 2 kg) were selected and randomly divided into four groups (18 in each group). Three replicate pens per group (six pigs per pen) were used, and two pigs were evaluated for each pen. The groups were fed the following diets: control group (CON), basic diet; chlorogenic acid group (CGA group), basic diet + 1 kg/ton *L. japonica* crude extract; Pueraria flavonoid group (PF group), basic diet + 1 kg/ton *Radix Puerariae* crude extract; and mix group (Mix group), basic diet + 0.5 kg/ton *L. japonica* crude extract + 0.5 kg/ton *Radix Puerariae* crude extract. The following results were obtained: (1) At the phylum level, Bacteroidetes, Firmicutes, Spirochaetes, Proteobacteria, Fibrobaeteres, and Kiritimatiellaeota were the main components of the fecal microbiota (top 5); the relative abundance of bacteria from phyla Firmicutes significantly increased in the Mix group than in the CON group (*p* < 0.05). At the genus level, Treponema_2, Rikenellaceae_RC9_gut_group, uncultured_bacterium_f_Lachnospiraceae, uncultured_bacterium_f_Prevotellaceae, and Prevotellaceae_NK3B31_group were the main components of the fecal microbiota (top 5); the relative abundance of bacteria from genus Lactobacillus significantly increased in the Mix group than in the CON group (*p* < 0.05). Chao1 and Ace counts were significantly higher in group CGA than in the CON group and group Mix (*p* < 0.05). The alpha and beta diversities and the relative abundance of fecal microbes were higher in all test groups than in the CON group. (2) The protein digestibility was significantly higher in the CGA and PF groups than in the CON group, and the TP digestibility was significantly higher in the CGA than in the CON and Mix groups (*p* < 0.05). In conclusion, *Lonicera japonica* and *Radix Puerariae* crude extract supplementation in the diet significantly changed fecal microbiota and improved the protein and TP digestibility of finishing pigs.

## 1. Introduction

Antibiotics are widely used to increase growth performance and disease resistance in livestock and poultry, but they have certain issues. In particular, pathogenic bacteria adapt to an antibiotic environment and develop resistance to antibiotics, resulting in death in humans [1]. Many countries have prohibited the addition of antibiotics to livestock feed while aiming at the serious problem of abuse of antibiotics in livestock and poultry breeding [2]. However, not adding antibiotics to the livestock diet results in a decrease in growth performance and feed conversion ratio, and an increase in treatment cost. Thus, finding a safe alternative to antibiotics is essential.

Honeysuckle is the dried flower bud or first flower of *Lonicera japonica* Thunb. (*L. japonica*) of the family Caprifoliaceae. It is a common Chinese medicine with strong antioxidant, anti-inflammatory, and antiviral effects [3]. Chlorogenic acid (CGA), also known as coffee tannic acid, is a phenolic acid formed by the condensation of caffeic acid and quinic acid [4]. CGA is widely found in natural plants, and its main sources are honeysuckle, eucommia leaves, coffee beans, and so forth. Among these, honeysuckle and eucommia leaves contain the highest content of CGA, which is the main antioxidant, antibacterial, and antiviral active ingredient [5,6,7].

*Pueraria lobata* is the dried root of *Radix Puerariae* (Willd.) Ohwi, is a leguminous plant with a long history of medicinal origin in China. It has been widely used for treating cardiovascular diseases, diabetes, Alzheimer’s, and cancer [8,9,10]. Pueraria flavonoid (PF) is a class of isoflavone derivatives extracted from *P. lobata*, mainly including puerarin, which has the functions of antioxidant, blood sugar reduction, and immunity regulation [11,12,13].

In the present study, we hypothesized that dietary supplementation with *L. japonica* and *Radix Puerariae* crude extracts might have a positive effect on the fecal microbiota and improve the apparent nutrient digestibility of finishing pigs. To test the hypothesis, the microbiota community composition of the fecal samples and the apparent nutrient digestibility were quantified, to evaluate the effects of *L. japonica* and *Radix Puerariae* crude extracts on fecal microbiota and apparent nutrient digestibility.

## 2. Materials and Methods

### 2.1. Experimental Design

The experiment was conducted in the piggery of the experimental base of Animal Husbandry and Veterinary Research Institute, Tianjin Academy of Agricultural Sciences. The tests were performed over a 50-day period. All the experimental procedures were approved by the Animal Ethics Committee of the Tianjin Academy of Agricultural Sciences.

A single-factor completely random design was conducted in this experiment. A total of 72 healthy Duroc × Landrace × Yorkshire crossbred barrows with 152 days of age and body weight of 93 ± 2 kg were purchased from Tianjin Nongkang Breeding Co., Ltd. (Tianjin, China) and randomly divided into four groups (18 in each group). Three replicate pens per group (six pigs per pen) were used. The groups were fed the following diets: control group, basic diet; CGA group, basic diet + 1 kg/ton *L. japonica* crude extract; PF group, basic diet + 1 kg/ton *Radix Puerariae* crude extract; and Mix group, basic diet + 0.5 kg/ton *L. japonica* crude extract + 0.5 kg/ton *Radix Puerariae* crude extract. The experiment was performed for 50 days.

*L. japonica* and *Radix Puerariae* crude extracts were purchased from Anhui Tianfulai Biotechnology Co., Ltd. (Lu’an, Anhui, China). The active component in the *L. japonica* crude extract and *Radix Puerariae* crude extract was 4.96% CGA and 93.6% PF, respectively, with no other nutrients.

### 2.2. Animal Management

The test hoggery adopted strict biosecurity measures to ensure full entry and exit of animals as well as complete isolation of breeders. The temperature in the test hoggery was controlled at 20–28 °C, with natural light and normal ventilation. The pigs were fed and watered freely during the trial period and the manure was cleaned daily. The trial pre-feeding period was 5 days, with transitional feeding by replacing the original diet with 20%, 40%, 60%, 80%, and 100% of the test diet. The formal experiment was conducted for 45 days, during which the health status of the test pigs (diarrhea or other diseases, death, and so on) was observed and recorded daily.

### 2.3. Experimental Diets

The experimental diets (Table 1) were prepared in accordance with the NRC (2012) standard. The feed samples in each group were collected, and the contents of crude protein, Ca, total P, and amino acids were determined. After the experiment was completed, the body weights of the pigs in each group were measured, and the total feed intake of the pigs in each pen was calculated. Moreover, average daily feed intake (ADFI), average daily gain (ADG), and feed conversion ratio (FCR) were measured.

### 2.4. Analysis of Bacterial Community and Data Analysis

On day 40 of the experiment, nine fecal samples were collected from each pen. The rectal feces were collected from the rectum using rectal swabs into sterile tubes, immediately transferred to liquid nitrogen for storage, and subsequently stored at −80 °C for further analysis. The samples were sent to Beijing Bemac Biotechnology Co., Ltd (Beijing, China). for 16s rRNA sequencing. The test was performed as follows.

#### 2.4.1. DNA Extraction and 16S rRNA Sequencing 

Bacterial DNA was extracted from fecal samples of the pigs using the Power Soil DNA Isolation Kit (MO BIO Laboratories, Carlsbad, CA, USA) following the manufacturer’s protocols. DNA quality and quantity were assessed by the ratios of 260 nm/280 nm and 260 nm/230 nm by Nano-Drop™ (Thermo Scientific, Waltham, MA, USA). Then, the DNA was stored at −80 °C until further processing.

The V3–V4 region of the bacterial 16S rRNA gene was amplified with a primer pair 338F* (Forward primer, 5’- ACTCCTACGGGAGGCAGCA-3’) and 806R (reverse primer, 5’-GGACTACHVGGGTWTCTAAT-3’). PCR amplification was performed in a total volume of 50 μL, including 10 µL of PCR buffer, 0.2 µL of Q5 High-Fidelity DNA polymerase, 10 µL of GC-rich enhancer, 1 µL of dNTP, 10µM of each primer, and 60 ng genomic DNA, made up with 50 µL of H_2_O.

The first-round PCR cycle conditions were as follows: a pre-denaturation at 95 °C for 5 min, followed by 15 cycles at 95 °C for 1 min, annealing at 50 °C for 1 min, 72 °C for 1 min, and extension at 72 °C for 7 min. The PCR products from the first step of the PCR were purified using VAHTS DNA clean beads (Vazyme, Nanjing, China). The second round of PCR was performed using the following thermal-cycling program: initial denaturation at 98 °C for 30 s, followed by 10 cycles of 98 °C for 10 s, 65 °C for 30 s, and 72 °C for 30 s, and a final extension at 72 °C for 5 min. All final PCR products were quantified using the Quant-iT dsDNA HS Reagent and were pooled. The samples were combined and purified for high-throughput sequencing analysis of bacterial rRNA genes using an Illumina Hiseq 2500 platform (PE250) from Biomarker Technologies Corporation, Beijing, China.

#### 2.4.2. Microbiota Data Analysis

The rarefaction curve was used to verify whether the amount of sequencing data was sufficient to reflect the diversity of species in the samples and to indirectly reflect the abundance of species in the samples [14]. A rarefaction curve of the Shannon index was plotted based on the Shannon index of each sample at different sequencing depths using Mothur software and R language tools. Operational taxonomic units (OTUs), that is, categorical operational units, were clustering OTU consistency at a similarity level of 97.0%.

Alpha and Beta diversity metrics were evaluated by QIIME2 version 2020.2 [15,16], and differential analysis among groups on alpha diversity metrics was processed by one-way ANOVA. Linear discriminant analysis (LDA) effect size (LEfSe) analyses were performed using the LEfSe tool [17], and LDA log-score threshold was set to 3.0 [18,19]. The metagenomes and function of the intestinal microbiota were analyzed using the PICRUSt2 (https://github.com/picrust/picrust2 accessed on 1 August 2022) and the differences in KEGG pathways between groups were analyzed by using STAMP (version 2.1.3) [20], the significance of the difference in function abundance between groups was evaluated by one-way ANOVA. Intestinal microbiome phenotype predictions were predicted with BugBase [21].

### 2.5. Determination of Apparent Digestibility

The feed samples were collected from each group, and the content of conventional nutrients in the feed samples was determined. The apparent digestibility of feed nutrients was determined by the acid-insoluble ash method using an endogenous indicator. The fecal samples were collected from each pen at 08:00–08:30, 11:00–11:30, 17:00–17:30, and 21:30–22:00 on the last 5 days of the test period, and about 400 g of feces was collected into one sealed bags per pen per day, then mixed with 20 mL of 4 mol/L hydrochloric acid, immediately frozen at −20 °C. It was used to determine the content of conventional nutrients in the feces of finishing pigs. The collected fecal samples were dried at 65 °C and then rewetted for 24 h. The samples were crushed using a grinder.

The total energy, crude protein, coarse fiber, crude fat, calcium, total phosphorus, and acid insoluble ash (AIA) contents in feed and feces were determined by the national standard methods. Among these, total energy was determined according to the international standard ISO 9831:1998 method using an oxygen bomb calorimeter 6400 Series (Parr Instrument Company, Moline, IL, USA). The crude protein, coarse fiber, crude fat, calcium, and total phosphorus contents were determined by the Kjeldahl method in GB/T 6432-2018 for crude protein, the filtration method in GB/T 6434-2006 for coarse fiber, GB/T 6433-2006 for crude fat, GB/T 6436-2018 for calcium, and spectrophotometry in GB/T 6437-2018 for total phosphorus in feed. The AIA content in feed and feces was determined with reference to GB/T 23742-2009.

The apparent total digestibility (ATTD) of nutrients was determined using the AIA method, calculated as ATTD (%)$=100-\left(\frac{M_{2n}}{M_{1n}}\times \frac{M_{1m}}{M_{2m}}\right)\times 100$ where *M*_1m_ is the AIA content in feed (%), *M*_2m_ is the AIA content in feces (%), *M*_1n_ is the nutrient content in feed (%), and *M*_2n_ is the nutrient content in feces (%).

### 2.6. Statistical Analysis

The experimental data were examined using a one-way ANOVA analysis of variance in SPSS Statistics 26.0 and multiple comparisons by the Duncan method. Data with *p <* 0.05 were considered significantly different. The values were expressed as mean ± standard deviation.

## 3. Results

### 3.1. Influences of L. japonica Crude Extract and Radix Puerariae Crude Extract on Growth Performance

During the experiment, the experimental pigs were healthy, and no death was recorded. Their growth performance is listed in Table 2. At the start of the experiment, the initial weights of the finishing pigs in different groups did not significantly vary. The final weights of the pigs in the experimental groups were not significantly different from that in the control group. The ADGs in the mix group was significantly higher than those in the control and CGA groups (*p* < 0.05). The ADFI in all the experimental groups increased to some extent compared to the control group (*p* > 0.05). The FCR in the mix group were significantly lower than those in the control group (*p* < 0.05).

### 3.2. Validity Analysis of Fecal Microbes

**Figure 1:**
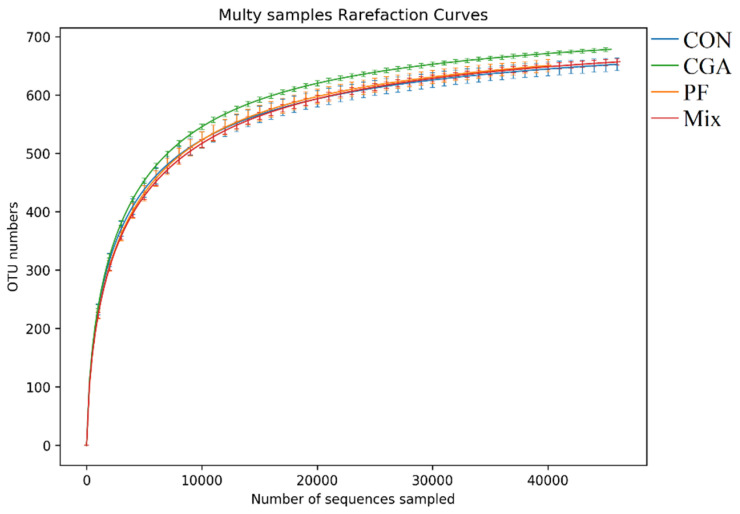
Rarefaction curves. The horizontal coordinate is the number of randomly selected sequencing strips, and the vertical coordinate is the number of features obtained based on the number of sequencing strips. Each curve represents one sample, marked with different colors.

The rarefaction curve was used to verify whether the amount of sequencing data was sufficient to reflect the diversity of species in the samples and to indirectly reflect the abundance of species in the samples [14]. As shown in Figure 1, the rate of emergence of new features (new species) in this experiment tended to level off under continuous sampling, demonstrating that the number of species in this experiment did not increase significantly with the number of sequencings and then the data were analyzed.

**Figure 2:**
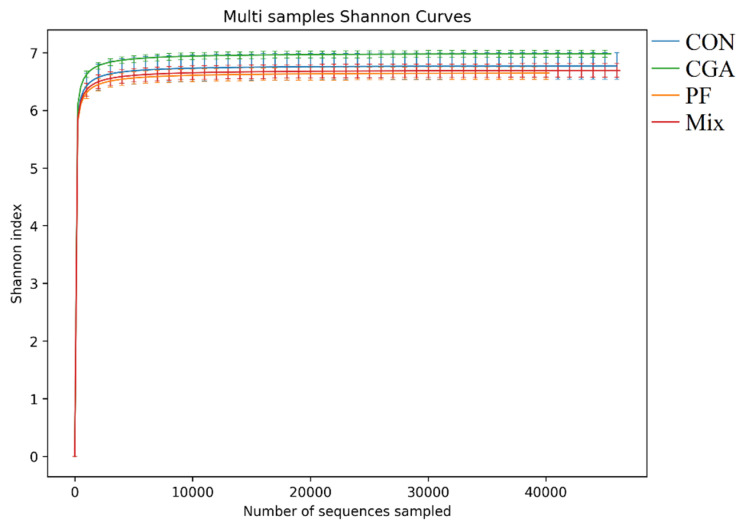
Rarefaction curve of the Shannon index. The horizontal coordinate is the number of sequencing strips randomly selected from a sample, and the vertical coordinate is the Shannon index. As the number of sequencing increased, more species were discovered until the species were saturated, and increasing the number of sampling strips did not reveal new features.

A rarefaction curve of the Shannon index was plotted based on the Shannon index of each sample at different sequencing depths using Mothur software and R language tools. The larger the Shannon index, the greater the number of species and the richer the species, indicating that most of the microbe species were covered in the samples. When the curve tended to be flat, it indicated that the amount of sequencing data was large enough so that the number of feature species did not increase with the amount of sequencing (Figure 2).

### 3.3. Operational Taxonomic Units of Rectal Microbes in Finishing Pigs

**Figure 3:**
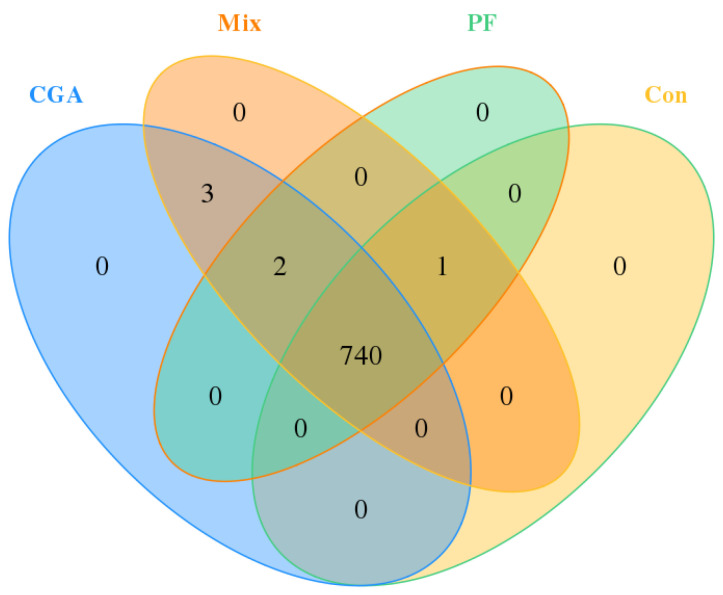
OTU Venn diagram of rectal microbes in finishing pigs. The overlapping numbers between multiple color graphs are the number of features shared between multiple samples, and the non-overlapping part is the number of unique features of each sample.

Operational taxonomic units (OTUs), that is, categorical operational units, were clustering OTU consistency at a similarity level of 97.0%. As shown in Figure 3, 746 OTUs were obtained after clustering the samples in each group, with 740 identical OTUs in the control and three experimental groups.

### 3.4. Alpha Diversity Analysis

**Figure 4:**
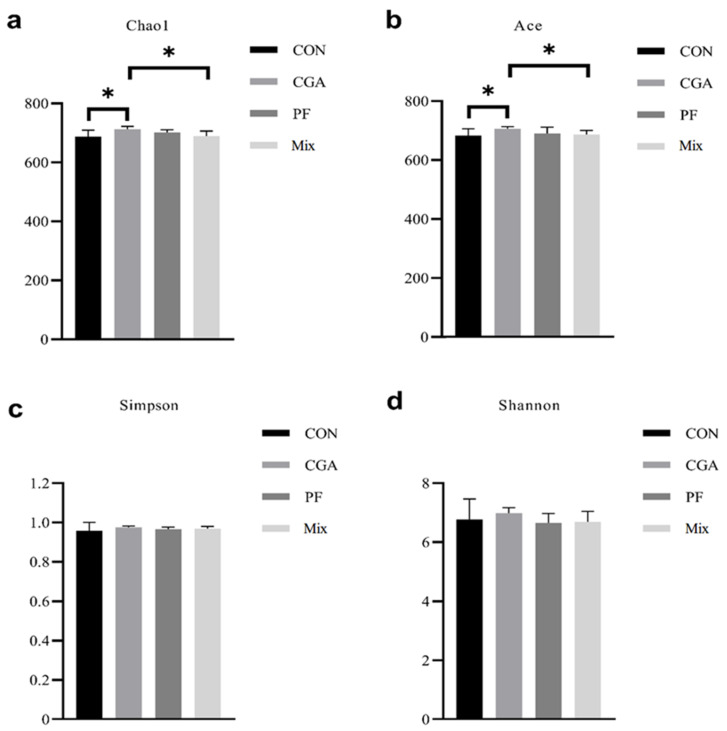
α diversity analysis of rectal microbes in finishing pigs. (**a**) Chao1 index, (**b**) Ace index, (**c**) Simpson index, and (**d**) Shannon index. All bar charts show means ± SD, * = *p* < 0.05, n = 9 in each group.

Alpha diversity reflects the abundance (richness) and species diversity (diversity) of an individual sample and is measured using multiple indicators: Chao1 index, Ace index, Simpson index, and Shannon index. Among these, Chao1 and Ace indexes can be used to measure species abundance, that is, the number of species. Simpson and Shannon indexes were used to measuring species diversity, with larger Simpson and Shannon indexes indicating higher species diversity in the samples [22]. As shown in Figure 4, Chao1 and Ace indexes were significantly higher in CGA than in the control group and Mix (*p* < 0.05). In comparison, Chao1 and Ace indexes were higher in PF and Mix than in the control group, although the difference was negligible (*p* > 0.05). The Simpson index in the three experimental groups was higher than that in the control group, although the difference was negligible (*p* > 0.05). The species abundance of the fecal microbiome in finishing pigs was higher in all three experimental groups than in the control group, and the fecal microbiome diversity was higher in CGA than that in the control group

### 3.5. Beta Diversity Analysis

**Figure 5:**
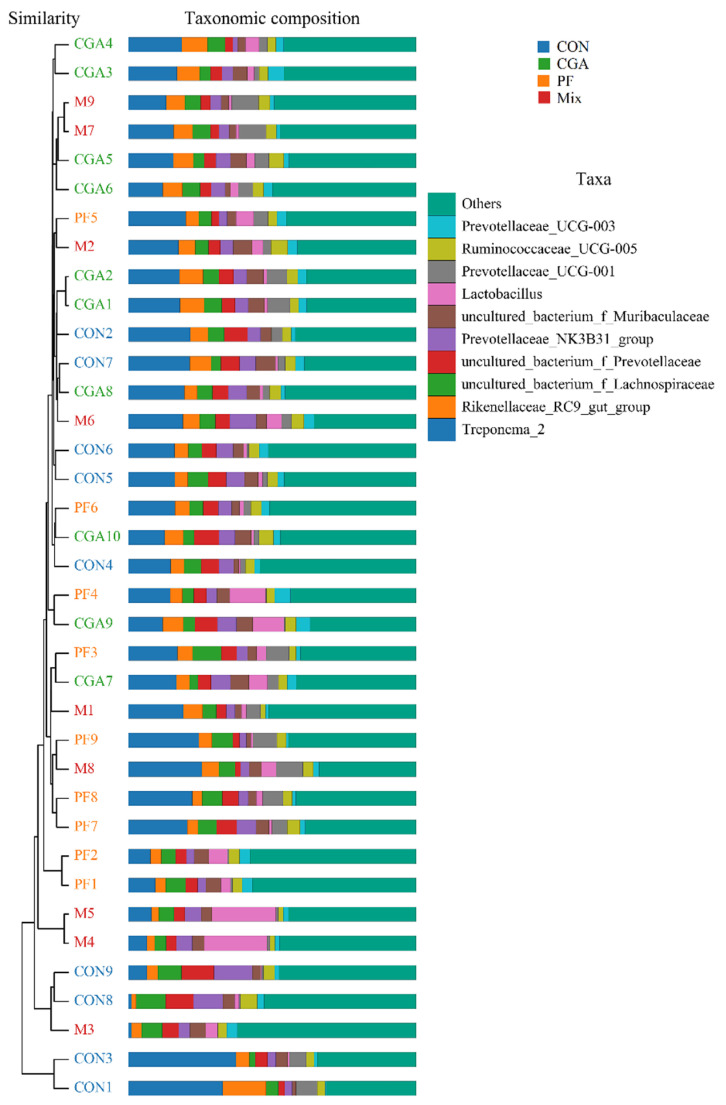
UPGMA clustering analysis of rectal microbe sin finishing pigs. The figure on the right represents the top 10 species according to the species abundance; the others are classified as Others, and those not annotated are classified as Unclassified.

Differences in the fecal microbiome in the three experimental groups were analyzed using the unweighted pair group method of the arithmetic mean (UPGMA) and nonmetric multidimensional scaling (NMDS). The UPGMA analysis was based on the four distance matrices obtained from beta diversity analysis, and the samples were clustered hierarchically. The closer the samples were to the sample clustering tree, the more similar the composition of the species of the two samples. As shown in Figure 5, the samples within the same test group were similar and the fecal microbiome was quite different among the three test groups, indicating that CGA and PF could influence the composition of the fecal microbiome in finishing pigs.

**Figure 6:**
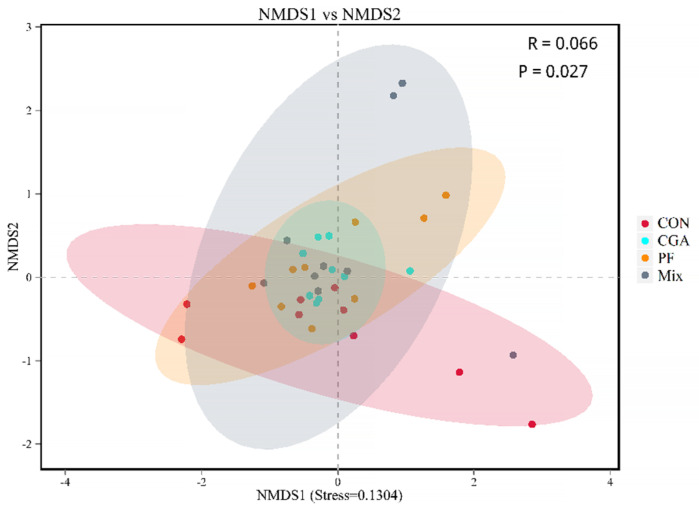
NMDS analysis of rectal microbes in finishing pigs.

NMDS analysis was performed on the samples, and points of the same color represented one sample from the same group. The closer the distance between the two points, the smaller the difference in community composition between them. As shown in Figure 6, the differences in the fecal microbiome community composition between the three experimental groups were small, but larger compared to that in the control group, indicating that CGA and PF had significant effects on fecal microbiome community composition.

### 3.6. Annotation and Taxonomic Analysis of Species

**Figure 7:**
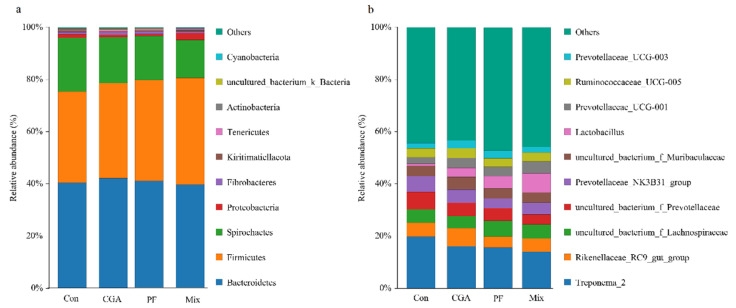
Annotation and taxonomic analysis of rectal species in finishing pigs. (**a**) Histogram of species distribution of phylum-level abundance (**b**) Histogram of species distribution of genus -level abundance. The horizontal coordinate is the sample name, and the vertical coordinate is the relative abundance (%).

Taxonomic annotation of feature sequences were processed by Bayesian classifier using SILVA as reference database [23]. Statistics on composition in each sample were calculated at the level of phylum, class, order, family, genus, and species. QIIME (Quantitative Insights into Microbial Ecology) was applied to obtain the abundance of each species in samples and distribution histogram at each taxonomic level were generated by certain R package. As shown in Figure 7a,b, the top ten fecal microbiomes in the three experimental groups of finishing pigs in terms of phylum-level abundance were as follows: Bacteroidetes, Firmicutes, Spirochaetes, Proteobacteria, Fibrobaeteres, Kiritimatiellaeota, Tenerieutes, Actinobactena, Uncultured_bacterium_k_Bacteria, and Cyanobacteria. As shown in Table 3, the abundance of Firmicutes in the Mix group was significantly higher than that in the control group (*p* < 0.05). As shown in Table 4, the top ten fecal microbiomes in finishing pigs in the three experimental groups in terms of genus-level abundance were as follows: *Treponema_2*, *Rikenellaceae_RC9_gut_group*, *uncultured_bacterium_f_Lachnospiraceae*, *uncultured_bacterium_f_Prevotellaceae*, *Prevotellaceae_NK3B31_group*, *uncultured_bacterium_f_Muribaculaceae*, *Lactobacillus*, *Prevotellaceae_UCG*−*001*, *Ruminococcaceae_UCG*−*005*, and *Prevotellaceae_UCG*−*003*.

As shown in Table 2 and Table 3, the abundance of Firmicutes in the three experimental groups was significantly higher than that in the control group (*p* < 0.05). The abundance of the *Rikenellaceae-RC9-gut-group* in the CGA group was significantly higher than that in the PF group (*p* < 0.05). The abundance of *uncultured-bacterium-f-Prevotellaceae* in the control group was significantly higher than that in the PF and control groups (*p* < 0.05). The abundance of the *Prevotellaceae-NK3B31-group* in the control group was significantly higher than that in the PF group (*p* < 0.05). The abundance of *Lactobacillus* in the Mix group was significantly higher than that in the control group (*p* < 0.05).

### 3.7. LEfSe Analysis of Different Groups

**Figure 8:**
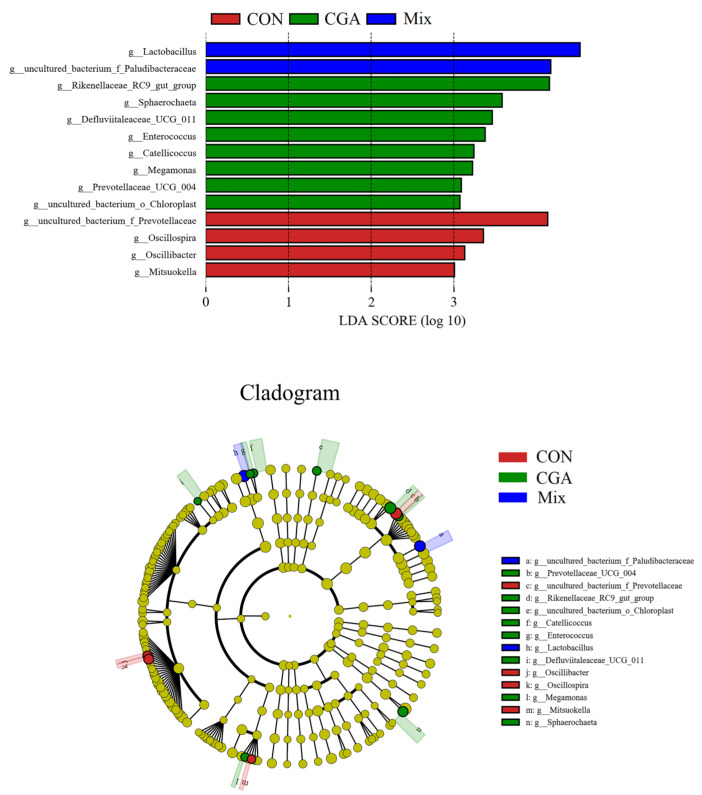
LDA distribution histogram and LEfSe cladogram. The radial circles of the cladogram represent the taxonomic levels from phylum to species. Each small circle at a different taxonomic level represents a taxon at that level, and the diameter of the small circles is proportional to the relative abundance. The coloring principle is to color the species with no significant difference in yellow, whereas the other species with differences are colored according to the group with the highest abundance. Different colors indicate different groups, and nodes with different colors indicate microbiotas that play an important role in the group represented by that color.

LEfSe (Line Discriminant Analysis (LDA) Effect Size) analysis is a nonparametric test (Kruskal–Wallis rank test) used to detect significant differences in abundance between subgroups in multiple samples, followed by LDA to classify the data and assess the impact of significantly different species (i.e., LDA score) [18]. The roles of microbiota in the three experimental groups were analyzed by counting the specimens with LDA score > 3.0, that is, biomarkers with statistically significant differences. In the cladogram, the species with no significant difference were uniformly colored in yellow, whereas the other species were colored according to the group with the highest abundance. Different colors represented different groups, and nodes with different colors indicated the microbiotas that played an important role in the group represented by the color. Figure 8 shows LDA distribution and LEfSe analysis of the cladogram. As observed, the microbiotas with LDA score > 3.0 in each group were as follows: *g_uncultured_bacterium_f_Prevotellaceae*, *g_Oscillospira, g_Oscillibacter*, and *g_Mitsuokella*. The important microbes in CGA were *g_Rikenellaceae_RC9_gut_group*, *g_Sphaerochaeta*, *g_Defluviitaleaceae_UCG_011*, *g_Enterococcus*, *g_Catellicoccus*, *g_Megamonas*, *g_Prevotellaceae_UCG_004*, and *g_uncultured_bacterium_o_Chloroplast*. The important microbes in Mix were *g_Lactobacillus* and *g_uncultured_bacterium_f Paludibacteraceae*.

### 3.8. Functional Gene Prediction Analysis

#### 3.8.1. PICRUSt2 Function Prediction

**Figure 9:**
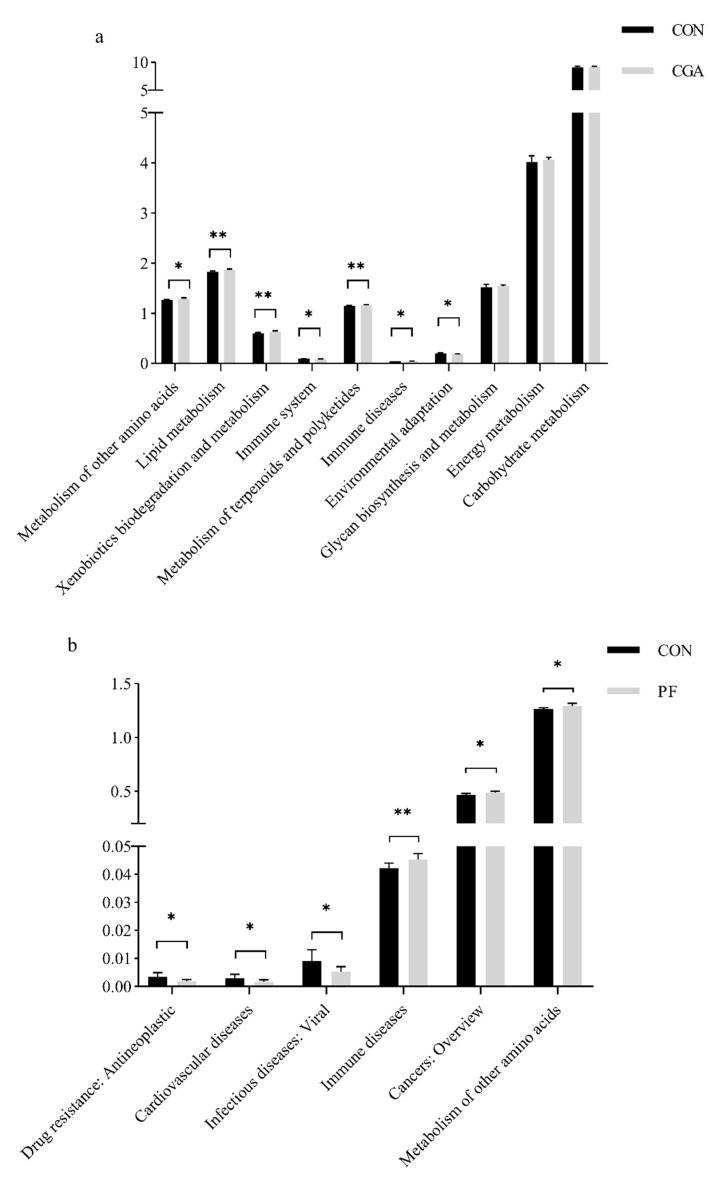

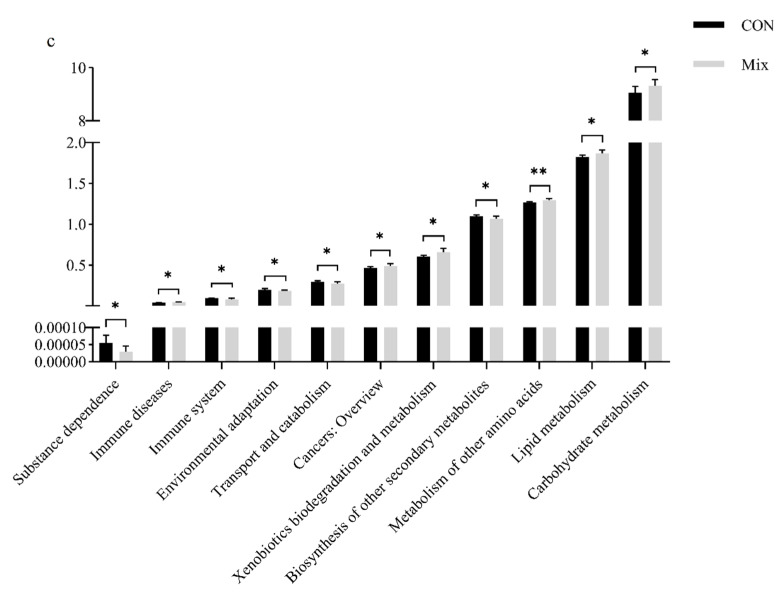
Composition and differential analysis of KEGG metabolic pathways. (**a**) Composition and differential analysis of the CON-CGA KEGG metabolic pathway. (**b**) Composition and differential analysis of the CON-PF KEGG metabolic pathway. (**c**) Composition and differential analysis of the CON-Mix KEGG metabolic pathway. All bar charts show means ± SD, * = *p* < 0.05; ** = *p* < 0.01.

PICRUSt2 was applied to perform species annotation on feature sequences based on reference phylogenetic tree. Potential functions and functional genes in samples were predicted based on Integrated Microbial Genomes (IMG) database, which further revealed the difference in functions between samples or groups [24]. As shown in Figure 9, lipid metabolism, metabolism of other amino acids, xenobiotics biodegradation and metabolism, and metabolism of terpenoids and polyketides in CGA were extremely significantly higher than those in the control group (*p* < 0.01). Moreover, the immune disease probability was significantly higher (*p* < 0.05). At the same time, the glycan biosynthesis and metabolism, energy metabolism, and carbohydrate metabolism were higher in CGA than in the control group, although the difference was negligible (*p* > 0.05). The immune system and environmental adaptation in CGA were significantly lower than those in the control group (*p* < 0.05). The probability of immune diseases (*p* < 0.01), metabolism of other amino acids, and cancers: overview (*p* < 0.05) were significantly higher in PF than in the control group. The probability of cardiovascular diseases, drug resistance: antineoplastic, and infectious diseases: viral was significantly lower in PF than in the control group (*p* < 0.05). However, the metabolism of other amino acids (*p* < 0.01), immune diseases, xenobiotics biodegradation and metabolism, carbohydrate metabolism, lipid metabolism, and cancers: overview (*p* < 0.05) were significantly higher in Mix than in the control group. However, the substance dependence, immune system, environmental adaptation, biosynthesis of other secondary metabolites, and transport and catabolism were significantly lower in Mix than in the control group (*p* < 0.05).

#### 3.8.2. Prediction of BugBase Phenotype

**Figure 10:**
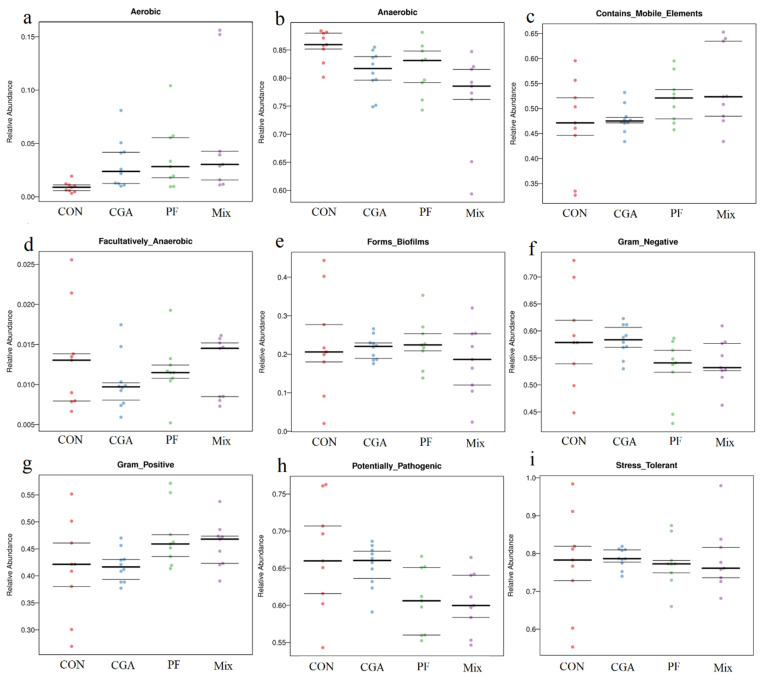
Prediction of BugBase phenotypes. The horizontal coordinate in the figure is the group name, the vertical coordinate is the relative abundance (%), and the three lines from bottom to top are the lower quartile, the mean, and the upper quartile, respectively, showing nine phenotypes: (**a**) aerobic, (**b**) anaerobic, (**c**) contains mobile elements, (**d**) facultatively anaerobic, (**e**) biofilms forms, (**f**) Gram-negative, (**g**) Gram-positive, (**h**) potentially pathogenic, and (**i**) stress tolerant.

BugBase is a novel method for analyzing complex microbiome data, which provides biologically relevant microbiome phenotype predictions at the organism level. First, BugBase normalizes OTU by predicted 16S copy number. Microbial phenotype is predicted based on given pre-calculated files [21]. *Lonicera japonica* and *Radix Puerariae* crude extracts improved the prediction of the fecal microbiome BugBase phenotype, with Mix aerobic, containing mobile elements significantly higher than the CON group (Figure 10a,c); anaerobic was significantly lower than the CON and PF (Figure 10b), and fecal microbiome potential pathogenicity significantly lower than CON group in Mix and PF (Figure 10h).

### 3.9. Effect of L. japonica and Radix Puerariae Crude Extracts on Intestinal ATTD in Finishing Pigs

The results of feed nutrient ATTD measurements are shown in Table 5. The crude protein digestibility in CGA and PF was significantly higher than that in the control group (*p* < 0.05). In comparison, Mix had slightly higher crude protein digestibility than the control group, but was not significantly different (*p* > 0.05). Total phosphorus digestibility was significantly higher in CGA than in the control group and Mix (*p* < 0.05), but not significantly different from that in PF (*p* > 0.05). Ca, crude fiber, crude fat, and overall digestibility showed an increasing trend but no significant difference in the three experimental groups compared with the control group.

## 4. Discussion

### 4.1. Effects of L. japonica Crude Extract and Radix Puerariae Crude Extract on the Production Performance of Finishing Pigs

As a green and healthy plant extract feed additive, CGA, has been added to the feed of finishing pigs [25], whereas PF is rarely added. The daily gain in weaned piglets can be significantly increased by adding 1000 mg/kg of CGA to their feed [26]. In our experiment, daily gain in finishing pigs increased, and the FCR decreased. These findings were possibly due to the *L. japonica* crude extract and *Radix Puerariae* crude extract can improve the gut microbial diversity of finishing pigs, enhancing the intestinal digestion and absorption of nutrients function.

### 4.2. Effects of L. japonica and Radix Puerariae Crude Extracts on the Fecal Microbiome in Finishing Pigs

The gastrointestinal tract of newborn animals is sterile before birth. After birth, some specific bacteria from the environment (*Streptococcus* and *Escherichia coli*) rapidly colonize the intestine, consuming oxygen. Later, anaerobic bacteria (e.g., *Lactobacillus* and *Bifidobacterium*) begin to colonize and form a specific microbial flora [27]. It was dominated by anaerobic bacteria and a few facultative anaerobes, including Firmicutes, Bacteroidete, Proteobacteria, Actinobacteria, Verrucomicrobia, and Fusobacteria, with Firmicutes and Bacteroidete as the dominant group, accounting for more than 90% of the total [28,29,30,31,32,33]. As the diet composition and living environment of piglets changed after weaning, the intestinal microbiome also changed, with *Bacteroides* spp. gradually becoming the dominant flora in the colon of adult pigs [34]. The intestinal barrier plays a crucial role in transporting nutrients and macromolecules and can block the entry of harmful macromolecules and microbes into the bloodstream, preventing endotoxemia and inhibiting the proliferation of pathogenic bacteria [31,35]. Yang et al. (2020) reported that the *L. japonica* extract (the main component was CGA) could enhance intestinal immune function and promote host health by modulating secretory immunoglobulins and cytokines [36]. Zhang et al. (2018) reported that adding CGA to feed for weaned piglets resulted in a significant increase in intestinal *Lactobacillus* [26]. Chen et al. (2019) found that the addition of 1000 mg/kg of CGA to the feed of weaned piglets resulted in a significant enhancement of microbial α-diversity in the cecum of piglets. At the phylum level, the relative abundance of Firmicutes and Bacteroidetes increased, and the relative abundance of Proteobacteria decreased. At the genus level, the relative abundance of *Lactobacillus* spp., *Proteus* spp., *Vibrio anaerobicus*, and *Heteroplasma* spp. Increased [37]. In this study, the top ten fecal microbiomes in finishing pigs in the three experimental groups in terms of phylum-level abundance were Bacteroidetes, Firmicutes, Spirochaetes, Proteobacteria, Fibrobaeteres, Kiritimatiellaeota, Tenerieutes, Actinobactena, Uncultured_bacterium_k_Bacteria, and Cyanobacteria. Among these, Bacteroidetes and Firmicutes showed an increasing trend. The top ten fecal microbiomes in finishing pigs in the three experimental groups in terms of genus-level abundance were *Treponema_2*, *Rikenellaceae_RC9_gut_group*, *uncultured_bacterium_f_Lachnospiraceae*, *uncultured_bacterium_f_Prevotellaceae*, *Prevotellaceae_NK3B31_group*, *uncultured_bacterium_f_Muribaculaceae*, *Lactobacillus*, *Prevotellaceae_UCG−001*, *Ruminococcaceae_UCG−005*, and *Prevotellaceae_UCG−003*. Among these, the abundance of Treponema_2 showed a downward trend, and the abundance of *Prevotellaceae_UCG−001* and *Prevotellaceae_UCG−003* showed an upward trend; the abundance of *Lactobacillus* in Mix was significantly higher than that in the control group. This was consistent with the results reported earlier, indicating that the addition of crude extracts of *L. japonica* and *Radix Puerariae* to the feed promoted the species diversity of rectal microbes in finishing pigs. They increased and decreased the relative abundance of probiotics and harmful bacteria, respectively. This was especially true when a mixture of *L. japonica* and *Radix Puerariae* crude extracts was added.

The gut microbiota can metabolize dietary and produces end products short-chain fatty acids (SCFAs) [38]. Meanwhile, SCFAs could regulate the abundance and composition of the gut microbiome, increase probiotics, inhibit the growth of pathogens, and improve intestinal barrier function. This was attributed to the fact that SCFAs could lower intestinal pH, and under acidic conditions, the proliferation of harmful bacteria (e.g., *E. coli*, Proteobacteria, and Spirochaetes) was inhibited, while the proliferation of probiotics (*Lactobacillus* and *Bifidobacterium*) was promoted [39,40,41,42,43,44]. In this study, *L. japonica* or *Radix Puerariae* crude extracts were added in the feed of finishing pigs. The results showed that the abundance of rectal Firmicutes and *Lactobacillus* in finishing pigs in the Mix group was significantly higher than that in the control group. However, the abundance of rectal Spirochetes and *Treponema pallidum* in the finishing pigs in the three experimental groups showed a downward trend. It might be due to the bacteriostatic effect of CGA and PF, which increased the abundance of Bacteroides and the content of SCFAs, and maintained the health of the gut microbiome, thus inhibiting the reproduction of harmful bacteria.

*Prevotella* was found to be the main carbohydrate-degrading bacterium in the gut, playing an important role in carbohydrate metabolism in the body [45,46]. The main metabolites of Bacteroidetes and Firmicutes were SCFAs and bile acids, which had the functions of energy supply, inhibition of pathogenic bacteria reproduction, maintenance of intestinal health, and participation in energy and lipid metabolism of the body [43,47,48]. Lagkouvardos et al. (2019) investigated bacterial diversity, ecology, functional potential, and phylogeny in the S24-7 family (Bacteroidetes) using 16S rRNA gene analysis and data from metagenomic, functional, and taxonomic studies of cultured species, and named it as Muribaculaceae. The members of Muribaculaceae were versatile in complex carbohydrate degradation [49]. In this study, the clustering analysis of the fecal microbiome and predictions of the KEGG metabolic pathway and BugBase phenotypes of rectal microbes in finishing pigs was executed. The results demonstrated that the abundance of Bacteroidetes, Firmicutes, *uncultured_bacterium_f_Muribaculaceae*, *Prevotellaceae_UCG−001* and *Prevotellaceae_UCG−003* in rectal microbes, lipid metabolism, other amino acid metabolism, polysaccharide biosynthesis and metabolism, energy metabolism, carbohydrate metabolism, exogenous substance biodegradation, terpenoid and polyketide metabolism, disease immunity, and other metabolic pathways in finishing pigs showed an increasing trend after adding *L. japonica* and *Radix Puerariae* crude extracts in the feed. BugBase phenotypes of fecal microbiome predicted decreased potential pathogenicity and increased stress resistance in each test group, indicating that crude extracts of *L. japonica* and *Pueraria lobata* could improve the structure of fecal microbiome and increase the abundance of rectal probiotics in finishing pigs, improve the metabolic pathways and functional phenotypes of the fecal microbiome in finishing pigs, and enhance the metabolism of nutrients by the fecal microbiome.

### 4.3. Effects of L. japonica and Radix Puerariae Crude Extracts on ATTD in Finishing Pigs

About 300 mg/kg eucommia extract (main component CGA) was added to the diet of yellow-feathered broiler. The results showed that the dry matter, crude protein content, and crude fat digestibility significantly improved, and the duodenal trypsin, amylase, and lipase activities significantly increased in the test group compared with the control group [50]. Zhang et al. (2018) found that the addition of 1000 mg/kg CGA to the feed of weaned piglets led to significantly enhanced duodenal intestinal villus height, V/C ratio, and abundance of duodenal lactobacilli [26]. The main function of the small intestine is to digest and absorb nutrients and water [51,52], thus, improved small intestine may enhance the absorption of nutrients such as CP and TP in the small intestine and improve digestibility. Chen et al. (2018) found that the addition of 1000 ng/kg of CGA to the feed of weaned piglets led to significantly enhanced contents of crude protein, crude fat, and ash ATTD, as well as the activity of alkaline phosphatase (AKP) in the jejunum [53]. AKP is a marker enzyme for primary digestion and absorption processes in the small intestine. There are reports that intestinal AKP plays an important role in maintaining intestinal nutrient absorption, mucosal mechanical, chemical, immune, and biological barriers [54]. Moreover, research shows that intestinal AKP is a gut mucosal defense factor maintained by enteral nutrition, and preserves the normal homeostasis of gut microbiota [55,56]. In this study, 1000 mg/kg of *L. japonica* crude extracts and 1000 mg/kg of *Radix Puerariae* crude extract were added to the feed of finishing pigs. The results showed that the TP digestibility was significantly higher in the CGA group compared with the control group. This might be because the regulatory effect of CGA improved intestinal AKP or other digestive enzymes activity and abundance of microorganisms related to phosphorus metabolism in the gut microbiota in the intestine of pigs (e.g., Actinobacteria), which promoted the digestion and absorption of nutrients in the feed for finishing pigs [57]. However, the underlying regulatory mechanisms still need to be further studied.

## 5. Conclusions

In conclusion, our findings showed that diets supplemented with *L. japonica* and *Radix Puerariae* crude extracts improved growth performance, abundance of beneficial bacteria in feces, and digestibility of CP and TP in finishing pigs. Thus, it was advantageous to use *L. japonica* and *Radix Puerariae* crude extracts as natural additives in the feed of finishing pigs.

## Figures and Tables

**Table 1:** Basic diet composition and nutrient level (air-dry basis).

Items	Contents
Corn, %	63.38
Wheat Bran, %	14.40
Soybean meal, %	13.00
Distillers dried grains with solubles (DDGS), %	5.0
CaHPO_4_, %	1.20
Limestone, %	0.90
NaCl, %	0.30
L-lysine hydrochloride (98.5%), %	0.27
L-threonine (97.5%), %	0.04
DL-methionine (99%), %	0.01
Choline chloride, %	0.10
Premix ^1,2^, %	1.40
Nutrient levels	
Dry matter, %	89.62
Gross energy (GE) ^3^, MJ/kg	16.63
Crude protein, %	16.09
Crude fiber, %	4.80
Ca, %	0.74
Total P, %	0.69
Lysine (Lys), %	0.90
Threonine (Thr), %	0.58
Methionine + Cysteine ^3^, %	0.63

^1^. Premix provides the following per kg of diet: 6500 IU of vitamin A, 2400 IU of vitamin D_3_, 20 mg of vitamin E, 2.4 mg of vitamin K_3_, 2.4 mg of vitamin B_1_, 6.6 mg of vitamin B_2_, 3 mg of vitamin B_6_, 0.025 mg of vitamin B_12_, 25 mg of nicotinic acid, 13 mg of pantothenic acid, 0.2 mg of biotin. ^2^. Premix provides the following per kg of diets: 15 mg of Cu, 150 mg of Fe, 80 mg of Zn, 50 mg of Mn, 0.6 mg of I, and 0.3 mg of Se. ^3^. Gross energy (GE) and Methionine + Cysteine were calculated, and other values were measured.

**Table 2:** Effects of *L. japonica* crude extract and *Radix Puerariae* crude extract on the growth performance of finishing pigs.

Items	Con	CGA	PF	Mix
Initial weight (kg)	94.44 ± 0.32	92.56 ± 0.75	93.28 ± 1.75	92.47 ± 0.97
Final weight (kg)	130.91 ± 1.09	129.67 ± 0.30	131.47 ± 2.27	131.94 ± 1.81
ADFI (kg)	3.00 ± 0.02	3.04 ± 0.03	3.03 ± 0.03	3.05 ± 0.03
ADG (kg)	0.83 ± 0.02 ^b^	0.87 ± 0.03 ^ab^	0.86 ± 0.02 ^ab^	0.90 ± 0.02 ^a^
FCR	3.63 ± 0.10 ^a^	3.50 ± 0.15 ^ab^	3.55 ± 0.12 ^ab^	3.40 ± 0.06 ^b^

Values in the same row with the same letter or without a letter in their superscripts are not significantly different (*p* > 0.05), whereas values in the same row with different letters in superscripts correspond to significant differences (*p* < 0.05).

**Table 3:** Annotation and taxonomic analysis of rectal species in finishing pigs (phylum level).

Item	CON	CGA	PF	Mix
Bacteroidetes	0.4162 ± 0.0623	0.4179 ± 0.0212	0.4059 ± 0.0526	0.3971 ± 0.028
Firmicutes	0.3533 ± 0.0623 ^b^	0.3665 ± 0.0163 ^ab^	0.3815 ± 0.0339 ^ab^	0.4072 ± 0.0557 ^a^
Spirochaetes	0.1943 ± 0.1141	0.1745 ± 0.0262	0.1788 ± 0.0551	0.1476 ± 0.0756
Proteobacteria	0.0114 ± 0.0115	0.0095 ± 0.0054	0.0080 ± 0.0033	0.0266 ± 0.0349
Fibrobacteres	0.0064 ± 0.0046	0.0082 ± 0.008	0.0084 ± 0.0089	0.0060 ± 0.0053
Kiritimatiellaeota	0.0055 ± 0.004	0.0050 ± 0.0019	0.0040 ± 0.0027	0.0045 ± 0.0022
Tenericutes	0.0051 ± 0.0051	0.0042 ± 0.0027	0.0044 ± 0.0017	0.0045 ± 0.0021
Actinobacteria	0.0033 ± 0.0019	0.0042 ± 0.0023	0.0034 ± 0.0015	0.0032 ± 0.0008
uncultured_bacterium_k_Bacteria	0.0009 ± 0.0013	0.0021 ± 0.0021	0.0015 ± 0.0012	0.001 ± 0.0011
Cyanobacteria	0.0007 ± 0.0006	0.0018 ± 0.001	0.0009 ± 0.0007	0.0006 ± 0.0003
Others	0.003 ± 0.0016	0.0026 ± 0.0009	0.0029 ± 0.0013	0.0019 ± 0.0006

Values in the same row with the same letter or no letter in superscripts are not significantly different (*p* > 0.05), whereas values in the same row with different letters in superscripts correspond to significant differences (*p* < 0.05).

**Table 4:** Annotation and taxonomic analysis of rectal species in finishing pigs (genus level).

Item	CON	CGA	PF	Mix
*Treponema-2*	0.19 ± 0.11	0.16 ± 0.03	0.17 ± 0.06	0.14 ± 0.08
*Rikenellaceae-RC9-gut-group*	0.06 ± 0.04 ^ab^	0.07 ± 0.02 ^a^	0.04 ± 0.01 ^b^	0.05 ± 0.02 ^ab^
*uncultured-bacterium-f-Lachnospiraceae*	0.06 ± 0.03	0.05 ± 0.01	0.06 ± 0.02	0.05 ± 0.01
*uncultured-bacterium-f-Prevotellaceae*	0.07 ± 0.03 ^a^	0.05 ± 0.02 ^ab^	0.05 ± 0.01 ^b^	0.04 ± 0.01 ^b^
*Prevotellaceae-NK3B31-group*	0.06 ± 0.03 ^a^	0.05 ± 0.02 ^ab^	0.04 ± 0.01 ^b^	0.05 ± 0.02 ^ab^
*uncultured-bacterium-f-Muribaculaceae*	0.04 ± 0.02	0.05 ± 0.01	0.04 ± 0.01	0.04 ± 0.01
*Lactobacillus*	0.01 ± 0.01 ^b^	0.03 ± 0.03 ^ab^	0.04 ± 0.04 ^ab^	0.07 ± 0.08 ^a^
*Prevotellaceae-UCG−001*	0.03 ± 0.02	0.04 ± 0.02	0.04 ± 0.03	0.05 ± 0.04
*Ruminococcaceae-UCG−005*	0.04 ± 0.01	0.04 ± 0.01	0.03 ± 0.01	0.03 ± 0.01
*Prevotellaceae-UCG−003*	0.02 ± 0.01	0.03 ± 0.01	0.03 ± 0.01	0.02 ± 0.01
*Others*	0.44 ± 0.08	0.43 ± 0.04	0.46 ± 0.07	0.46 ± 0.09

Values in the same row with the same letter or no letter in superscripts are not significantly different (*p* > 0.05), whereas values in the same row with different letters in superscripts correspond to significant differences (*p* < 0.05).

**Table 5:** Effect of crude extract of honeysuckle and *Pueraria lobata* root on the apparent digestibility of nutrients in finishing pigs.

Items	CON	CGA	PF	Mix
Crude protein (CP)	86.62 ± 0.99 ^b^	88.07 ± 1.61 ^a^	88.11 ± 1.07 ^a^	87.5 ± 0.62 ^ab^
Crude fiber (CF)	46.23 ± 4.23	46.70 ± 3.82	46.42 ± 3.44	46.50 ± 2.97
Ether extract (EE)	74.37 ± 1.76	75.14 ± 1.69	75.65 ± 1.58	74.97 ± 1.57
Ca	57.41 ± 5.76	57.38 ± 3.27	56.89 ± 1.95	58.95 ± 6.11
Total phosphorus (TP)	55.84 ± 2.71 ^b^	59.51 ± 1.38 ^a^	58.50 ± 2.37 ^ab^	56.39 ± 2.39 ^b^
Gross energy (GE)	88.54 ±0.94	88.8 ± 0.52	88.52 ± 0.77	88.60 ± 0.55

Values in the same row with the same letter or no letter in superscripts are not significantly different (*p* > 0.05), whereas values in the same row with different letters in superscripts correspond to significant differences (*p* < 0.05).

## Data Availability

The datasets analyzed in the current study are available from the corresponding author on reasonable request.
